# What can visual electrophysiology tell about possible visual-field defects in paediatric patients

**DOI:** 10.1038/s41433-021-01680-1

**Published:** 2021-07-16

**Authors:** Siân E. Handley, Maja Šuštar, Manca Tekavčič Pompe

**Affiliations:** 1 UCL Great Ormond Street Institute of Child Health, London, UK; 2 Clinical and Academic Department of Ophthalmology, Great Ormond Street Hospital for Children NHS foundation trust, London, UK; 3 Unit for Visual Electrophysiology and Paediatric Ophthalmology Department, Eye Hospital, University Medical Centre Ljubljana, Ljubljana, Slovenia

**Keywords:** Eye manifestations, Object vision, Pattern vision

## Abstract

Recognising a potential visual-field (VF) defect in paediatric patients might be challenging, especially in children before the age of 5 years and those with developmental delay or intellectual disability. Visual electrophysiological testing is an objective and non-invasive technique for evaluation of visual function in paediatric patients, which can characterise the location of dysfunction and differentiate between disorders of the retina, optic nerve and visual pathway. The recording of electroretinography (ERG) and visual-evoked potentials (VEP) is possible from early days of life and requires no subjective input from the patient. As the origins of ERG and VEP tests are known, the pattern of electrophysiological changes can provide information about the VF of a child unable to perform accurate perimetry. This review summarises previously published electrophysiological findings in several common types of VF defects that can be found in paediatric patients (generalised VF defect, peripheral VF loss, central scotoma, bi-temporal hemianopia, altitudinal VF defect, quadrantanopia and homonymous hemianopia). It also shares experience on using electrophysiological testing as additional functional evidence to other tests in the clinical challenge of diagnosing or excluding VF defects in complex paediatric patients. Each type of VF defect is illustrated with one or two clinical cases.

## 摘要

在儿童患者中识别潜在的视野 (VF) 缺损可能具有挑战性, 尤其是针对5岁之前和发育迟缓或智力残疾的儿童。视觉电生理检查是一种客观、无创评价儿童视功能的技术, 它能明确儿童视功能异常的部位, 并能辨别视网膜、视神经和视觉通路的异常。视网膜电流图 (ERG) 和视觉诱发电位 (VEP) 的记录可以从生命早期开始, 与患者的主观输入关系不大。由于ERG和VEP检查的起源是已知的, 所以电生理变化的模式可以为那些不能进行精确视野检查的儿童提供视野的相关信息。这篇综述总结了以前发表的几种常见类型的视野缺损的电生理学结果, 这些缺损可在儿童患者中发现 (包括全部视野缺损、周围视野丢失、中心暗点、双颞侧偏盲、垂直性视野缺损、象限性偏盲和同侧偏盲) 。并分享了使用电生理检查作为其他检查辅助证据的经验, 以解决诊断或排除复杂儿童患者视野缺损的临床挑战。每种类型的视野缺损都会用一到两个临床病例来阐释。

## Introduction

Visual-field (VF) testing is an important part of the assessment of children and infants in the paediatric ophthalmology clinic. Perimetry has been shown to be possible in some children without learning disability as early as 5 years old and to the same standard of an adult at around the ages of 10 to 12 years old [1]. However, developmental delay, intellectual disability and autistic spectrum disorder are found within an array of visual deficits (both anterior pathway and cortical) that present in the first few years of life [2]. This means there are children that need an assessment of their VF where formal perimetry is not achievable. Many researchers have recognised this problem and developed new perimeters that reduce the need for accurate subjective responses from the child by utilising eye movements [3, 4]. The Saccadic Vector Optokinetic Perimeter (SVOP) is one such device and was successfully tested in 75% of a cohort of children with brain tumours (sensitivity of 100% and specificity of 50%), 12% in a cohort of children with neurodisability and 62.5% in a cohort of normal children with field loss [3, 5]. In clinical practice when formal visual fields cannot be undertaken in a child, visual fields to confrontation are undertaken looking for the child’s behavioural responses. This is limited by the child’s attention, co-operation and learnt compensatory eye movements. As a result confrontation visual fields are generally believed to have a low sensitivity [4, 6].

Optical coherence tomography (OCT) has been suggested to be easier to perform in children than visual fields and has the added advantage of being possible to capture under anaesthesia [7]. It has been shown to be able to detect neural and retinal causes of visual-field loss and in turn infer visual-field loss [7–9]. However, this structure and function relationship is discordant and may not always be detected simultaneously. This appears to be dependant on the stage, location and mechanism of the pathology. For example in glaucoma OCT has been shown to detect structural glaucomatous changes before the functional change is detected on perimetry and as a result is preferred in early disease yet is limited by a floor effect in later disease as the retinal nerve fibre layer becomes so thin [10]. In retinitis pigmentosa (RP) OCT scans have shown to correlate well to preserved central visual function [9]. Damage to the visual pathway causes trans-synaptic degeneration (TSD), which can be detected in the eye using OCT [11, 12]. However, the time course to this degeneration that has been demonstrated in adults has been shown to have an age effect [11]. This means the time between visual pathway insult and its evidence being detectable on OCT in the eye of a child is less understood, which is important to consider in the monitoring of visual pathway tumours. In traumatic optic neuropathy, OCT changes have been reported to be detectable quicker at 2–3 weeks post injury but it is unclear if the rate of change is the same in optic nerve glioma [13]. Gu et al. [13] studied children with optic pathway gliomas and found OCT could differentiate between eyes with and without vision loss with 88.9% of those with decreased macular ganglion cell complex thickness having vision loss.

In clinical practice many complex paediatric patients are referred for visual electrophysiology testing to look for additional functional evidence to add to the diagnosis or exclusion of a possible VF defect. Visual electrodiagnostic tests are extensively used in establishing the diagnosis and in the monitoring of the paediatric patient’s condition. Combined recording of electroretinogram (ERG) and visual-evoked potential (VEP) can functionally dissect the visual pathway and identify the site of dysfunction in the paediatric patients visual pathway, whether the retina, optic nerve or visual cortex are the primary reason for vision loss [14, 15]. Recording of the VEP and ERG is possible from the first day of life and requires no subjective input from the patient. The International Society for Clinical Electrophysiology of Vision (ISCEV) standards recognise that adult-based tests need adaptation for successful implementation in paediatric practice, for example the use of abbreviated protocols to record an electroretinogram or the combination of tests to fully assess the paediatric patient [16]. However, as with any clinical test there are factors that affect the acquisition and interpretation of the data and in turn what diagnostic information can be acquired about the VF within this challenging group.

This review shares experience and illustratory cases from two tertiary paediatric centres, demonstrating when visual electrodiagnostic tests can (and cannot) provide evidence to the clinical challenge of supporting or excluding the diagnosis of VF defects in paediatric ophthalmology patients.

## Methodology

Clinical electrophysiological testing of the visual system is based on non-invasive tests that provide an objective assessment of the visual system function. The tests are performed according to the standards of the International Society for Clinical Electrophysiology of Vision (ISCEV), which has published the guidelines for the following methods. Electrooculography [17] is a method for evaluation of functional integrity of the retinal pigment epithelium (RPE) and its interaction with the outer retina. Full-field ERG (ffERG) [18] is a generalised electric response of the retina, which evaluates the function of peripheral retina. With a combination of responses, elicited after the dark-adapted (DA) and light-adapted (LA) conditions, it determines whether the rod system or the cone system is more affected, or whether the dysfunction appears at the level of photoreceptors or post-photoreceptoraly. Multifocal ERG (mfERG) [19] evaluates the function of multiple discrete areas of the central retina. Similarly, also the pattern ERG (PERG) [20] evaluates the function within the macular region, but it emerges largely from the ganglion cells (N95 component), with a contribution from the photoreceptors and corresponding bipolar cells (P50 component). Visual-evoked potentials (VEP) [21] can be recorded either by a pattern-reversal, pattern-onset or flash stimuli and assess the conduction along the optic nerves and the functional integrity of the visual pathway up to the primary visual cortex. Furthermore, with the use of several recording electrodes over the occipital lobe of the visual cortex (i.e., the multichannel VEP) and use different types of stimulation (full-field and half-field stimuli), the VEP can also localise the impairment along the visual pathway (optic nerve, chiasm, retrochiasmal visual pathway or visual cortex).This is further discussed in the relevant sections.

Electrophysiological assessment in paediatric patients can be performed with the above-mentioned standard methods, but only in older children that are able to follow strict recording procedures. In infants, young children and paediatric patients with disabilities, the recording requires customised methods and well-trained staff. There is a long history of successful electrophysiological evaluation of the visual pathway in paediatric patients in specialist centres worldwide. The protocol used at Great Ormond Street Hospital, London, UK is described in each edition of Taylor and Hoyt’s Paediatric Ophthalmology and Strabismus [15]. An adaption that has been adopted by both our teritiary paediatric centres is, in brief, that flash ERGs are recorded with skin electrodes, non-dilated pupils and no formal dark adaptation. The rod and cone contributions are separated by scotopic blue vs. red and 30 Hz flashes. Compared to the ISCEV standard this adapted ERG technique shows excellent overall sensitvity of 95% (accuracy 86%) [22]. VEPs to pattern-reversal, pattern-onset and flash stimuli are recorded, stimulation protocols are adapted according to individual need. During a recording, distraction with cartoon, music, audio books and noisy toys is used. The attention and gaze for a successful recording are directed and encouraged by interaction and play. Such methodology is in use in both tertiary paediatric centres that contributed to the current work. The testing protocols are undertaken by personnel who are very experienced in tertiary paediatric practice working in pairs, therefore it may be less feasible in other settings. In this review the visual pathway causes of visual-field loss were divided in to sections based on the classification by Wirtschafter and Walsh [23]. Key words were derived from the classified visual-field loss and electrophysiological techniques. A key word and MeSH term search of PubMed/MEDLINE was undertaken to retrieve papers with no date range restrictions. Results were hand searched for those relating to paediatric patients/participants. References list of selected papers were checked for additional relevant works. Design of this review and contribution of exemplary cases followed the tenets of the Declaration of Helsinki.

## Complete visual-field loss

The causes of complete or generalised VF loss can be divided into three categories. The first group of disorders causing generalised VF defects are pre-retinal, including ptosis of the eyelid, cataract, vitreous haemorrhage and other media opacities. Visual electrodiagnostics can be of great help to the clinician in such cases. ERGs and VEPs, particularly to flash stimulation, can offer useful information of preserved function when ocular opacities are present [24].

The second group of disorders causing generalised VF defects are retinal causes, where diffuse photoreceptor pathology affects the visual-field profoundly. Leber congenital amaurosis (LCA) is a group of monogenic inherited retinal degenerations that typically show early-onset and severe visual dysfunction, it represents approximately 15 % of congenital blindness. LCA is mainly recessively inherited and manifests with signs of very poor visual function and roving eye movements or nystagmus from birth. Eye-poking or eye-rubbing, the oculodigital sign, may be present and could eventually lead to sunken orbits, cataract and keratoconus. The majority of patients have normal fundi at presentation, but disc pallor, vessel attenuation and pigmentary changes may follow [25]. In view of the potential for normal fundus appearance at presentation the combination of clinical and electrophysiological investigation is an essential part of establishing the diagnosis of LCA. The flash ERG is typically severely reduced or undetectable from early infancy, but sometimes attenuated flash VEP could be detected even though the flash ERG is absent [26]. Children with LCA and a detectable flash VEP typically have very poor vision, limited to perception of light or hand movements. In order to detect different subtypes of LCA that may benefit from gene therapy in the future, thorough electrophysiological work-up in combination with clinical examination and genetic testing will be essential [27]. Other early-onset retinal dystrophies can give a similar clinical picture as LCA. However, the visual function in other early-onset retinal dystrophies is usually better, depending on the underlying gene and mutation [25] and may also be part of a syndromic presentation. Advanced stages of retinopathy of prematurity (ROP) can also result in low visual acuity or even blindness due to retinal detachment. Both the rods [28] and the cones [29] are known to be affected in advanced ROP.

The third group of disorders that can cause generalised unilateral or bilateral VF defects are conditions related to optic nerve and post retinal pathology. A broad group of optic neuropathies can be divided according to the cause into demyelinating, inflammation related (but not demyelinating), vascular (ischaemic), toxic, nutritional, radiation, infiltrative, compressive, paraneoplastic, traumatic, hereditary and developmental. The visual-field defects in optic neuropathies can take several patterns, including central, altitudinal, arcuate, and diffuse defects. The pattern of visual-field defect is not specific to any aetiology and almost any type of field defect can occur with any optic neuropathy. Diffuse visual-field defects represent about half of the visual-field defects in optic neuritis [30]. Optic nerve hypoplasia is a possible cause for poor vision or even blindness from infancy as such requiring electrophysiological assessment. The VEP might show a varying degree of abnormality and might be undetectable in severe cases, while ERGs are typically normal [14]. The degree of fVEP attenuation and horizontal optic disc diameter to the disc-macula distance have been shown to be important parameters of visual acuity prediction in babies [31]. Furthermore, children’s vision can be severely affected by damage to the visual pathways in the cortical and sub-cortical regions, as seen in cerebral visual impairment (CVI), which can in its most severe form cause complete blindness. CVI may be caused by hypoxic birth injury, trauma, occipital lobe infarction and other causes. CVI can be electrophysiologically confirmed with normal flash ERG and abnormal flash VEP in severe cases [14]. However, the interpretation of the residual flash VEP in the most severe cases of CVI should be made cautiously, as the VEP might be contaminated with an artefact caused by ERG activity. Simultaneous recording of the flash ERG with the flash VEP allows the differentiation of this artefact from true post-retinal activation [32].

Two cases of generalised visual-field loss are presented, binocular loss in a girl with LCA and monocular loss in a boy with optic nerve hypoplasia.

### Case 1: Bilateral blindness due to early retinopathy

**Fig. 1:**
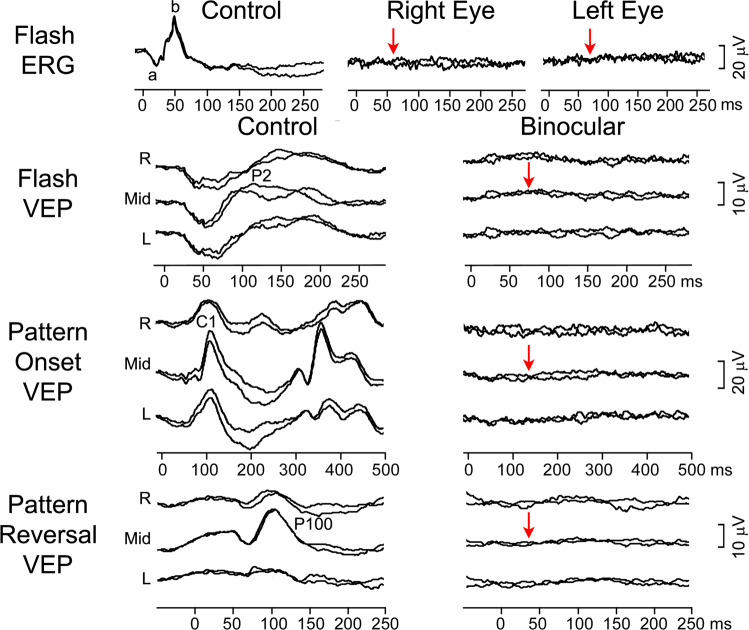
Electrophysiological findings from case 1: a girl with poor eye contact and roving eye movements. Red arrows indicate severely reduced to undetectable ERG bilaterally and VEP responses to binocular stimulation. The main components of the ERG (a and b wave), flash VEP (P2), pattern-onset VEP (C1) and pattern-reversal VEP (P100) are labelled.

A 15-month-old girl was referred to the paediatric ophthalmologist for a second opinion. At 3 months, her mother noticed she was not able to maintain eye contact and was not following objects. Previously glasses with high-hyperopic correction were prescribed. At the eye examination, roving eye movements and oculodigital reflex were noticed, she was not able to fix and follow. Fundus examination revealed pale optic discs bilaterally. Visual electrophysiology was carried out (Fig.  1). The flash ERG was undetectable from both eyes and the VEP to binocular stimulation with flash, pattern-reversal and pattern-onset stimuli was undetectable as well. These findings were in keeping with her visual behaviour and showed that a generalised retinal dysfunction was the cause. The clinical and electrophysiological findings let to a diagnosis of Leber’s congenital amaurosis.

### Case 2: Unilateral blindness due to optic nerve hypoplasia

**Fig. 2:**
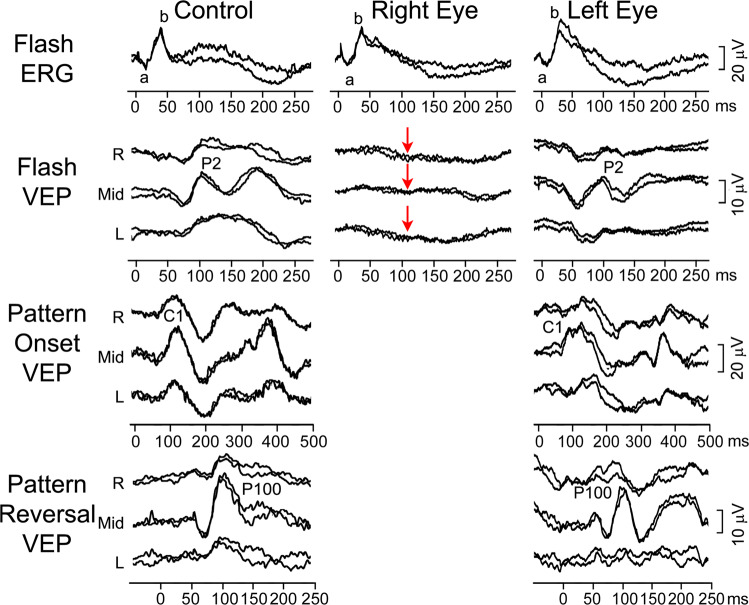
Electrophysiological findings from case 2: a boy with right optic nerve hypoplasia. Red arrows indicate severely reduced to undetectable flash VEP responses, while the ERG was normal in the right eye. R—right occiput, Mid—mid-occiput, L—left occiput. The main components of the ERG (a and b wave), flash VEP (P2), pattern-onset VEP (C1) and pattern-reversal VEP (P100) are labelled.

A 3-year-old boy was referred for clinical and electrophysiological examination due to optic nerve hypoplasia of his right eye (RE). His co-operation at the eye examination was poor, and he could not reliably perform visual acuity testing. Fundus examination, showed his RE optic disc was extremely small, markedly hypoplastic and pale with a ring of peripapillary atrophy, whereas the optic disc in his left eye (LE) appeared normal. He also had markedly reduced direct pupillary response on his RE. Electrophysiologically (Fig.  2), the flash ERG showed he had normal retinal function of both eyes. The VEP of the RE was non-detectable indicating a marked post-retinal abnormality while the VEP of the LE was normal. Based on the appearance of the right optic nerve and electrophysiological findings poor vision of his RE was postulated, probably within light perception range.

## Peripheral visual-field defect

Peripheral VF defect or constriction can appear as monocular or bilateral defect, as a consequence of various hereditary, inflammatory, autoimmune and toxic retinal disorders, or due to abnormalities at the optic disc, such as glaucoma or optic nerve head swelling. Constricted VF can result also from media opacities or deliberate malingering, while localised peripheral field abnormalities can result from occlusion with nose and upper eyelid [23].

Peripheral VF constriction is a typical feature of retinitis pigmentosa (RP) a rod cone dystrophy associated with severe functional abnormality of the peripheral retina. Rod mediated ffERG responses are either severely abnormal or extinguished [33]. Several studies have shown the amplitude of the ffERG is proportional to the area of functional retina in patients with RP with the reported correlation ranging from *p* ≤ 0.04 to *p* ≤ 0.001 [34–37], more severe constriction of the VF resulting in smaller amplitude ffERG. It is estimated RP patients lose 16–18.5% of ERG amplitude with disease progression [9, 37] and so visual-field progression can be inferred from the changing ffERG. As the VF constriction can occur early in disease course and might not be accompanied with typical intraretinal pigment deposition, ERG testing is particularly important in children [15]. On the other hand, preserved PERG and pattern-reversal VEP might show the extent of central VF preservation. Both PERG, as well as the pattern-reversal VEP reflect the activity arising from the central VF [38, 39]. Thus, Robson et al. [40] used the pattern ERG as an objective and reproducible index of macular preservation in patients with RP by changing the field size of the presented pattern stimuli. They found the PERG P50 amplitude correlated with the ring of autofluorescence (*r* = 0.80, *p* < 0.0005, *n* = 30). This ring of autofluorescence and the inner/outer segment boundary on OCT have also been found to also correspond well both with each other with kinetic perimetry of the functional visual-field in adult patients with RP [9, 41, 42].

Paediatric patients with refractory epilepsy treated with vigabatrin might develop bilateral concentric constriction of the VF, that typically exhibits a binasal annular defect within the central 30° and relative temporal sparing [43]. Adverse visual effects and retinal toxicity, found in those patients0is commonly associated with ERG abnormalities. The cone system is predominantly affected, which can be recognised from reduced light-adapted ERG and 30-Hz flicker ERG, reduced d-wave amplitude of the On-Off ERG and abnormalities in oscillatory potentials [44–49]. Some of these changes are reversible with cessation of the therapy [47, 48], and where medically appropriate the ERG can be used to detect retinal changes in pre-perimetric children on vigabatrin therapy.

While retinal causes of the peripheral VF loss can be simply determined by ffERG, the identification of post retinal causes is more challenging. The P100 wave of the pattern-reversal VEP, which is the most precise measure of the post retinal functional integrity [16], emerges from the central VF [38] and is largely insensitive for detection of peripheral field loss [50]. On the other hand, multifocal VEP (mfVEP) technique allows assessment of a much larger cross-sectional area of the optic nerve [51] and can detect clear abnormality in case of peripheral field loss [50]. Although multifocal VEP technique can be a demanding test, it was shown to be possible in children from 5 years of age and might represent a promising objective test of children’s visual fields even before they are able to perform subjective VF testing [52, 53]. Furthermore, Harding et al. [54, 55] developed field-specific visual-evoked potentials for identifying VF defects in children treated with vigabatrin and unable to perform perimetry. The pattern-reversal VEP-based technique consisted of central (0°–5° radius) and peripheral stimulus (30°–60° radius) that increased in size with eccentricity. The electrodiagnostic tests were more achievable in the participants (VEP *n* = 35/39 ERG *n* = 26/39) than perimetry (*n* = 12/39) [55]. The field-specific VEP had a sensitivity of 75% and a specificity of 87.5% [55]. This technique was further developed by Hébert-Lalonde et al. [56, 57] that used steady-state VEP and pattern ERG to field-specific radial checkerboards. They showed significantly lower responses to peripheral stimulations in the vigabatrin-exposed group, that were negatively correlated to vigabatrin exposure duration.

The following two cases depict the use of visual electrophysiology in determining either the extent or the cause of peripheral visual-field abnormality in children.

### Case 3: Constricted visual fields due to retinal dystrophy

**Fig. 3:**
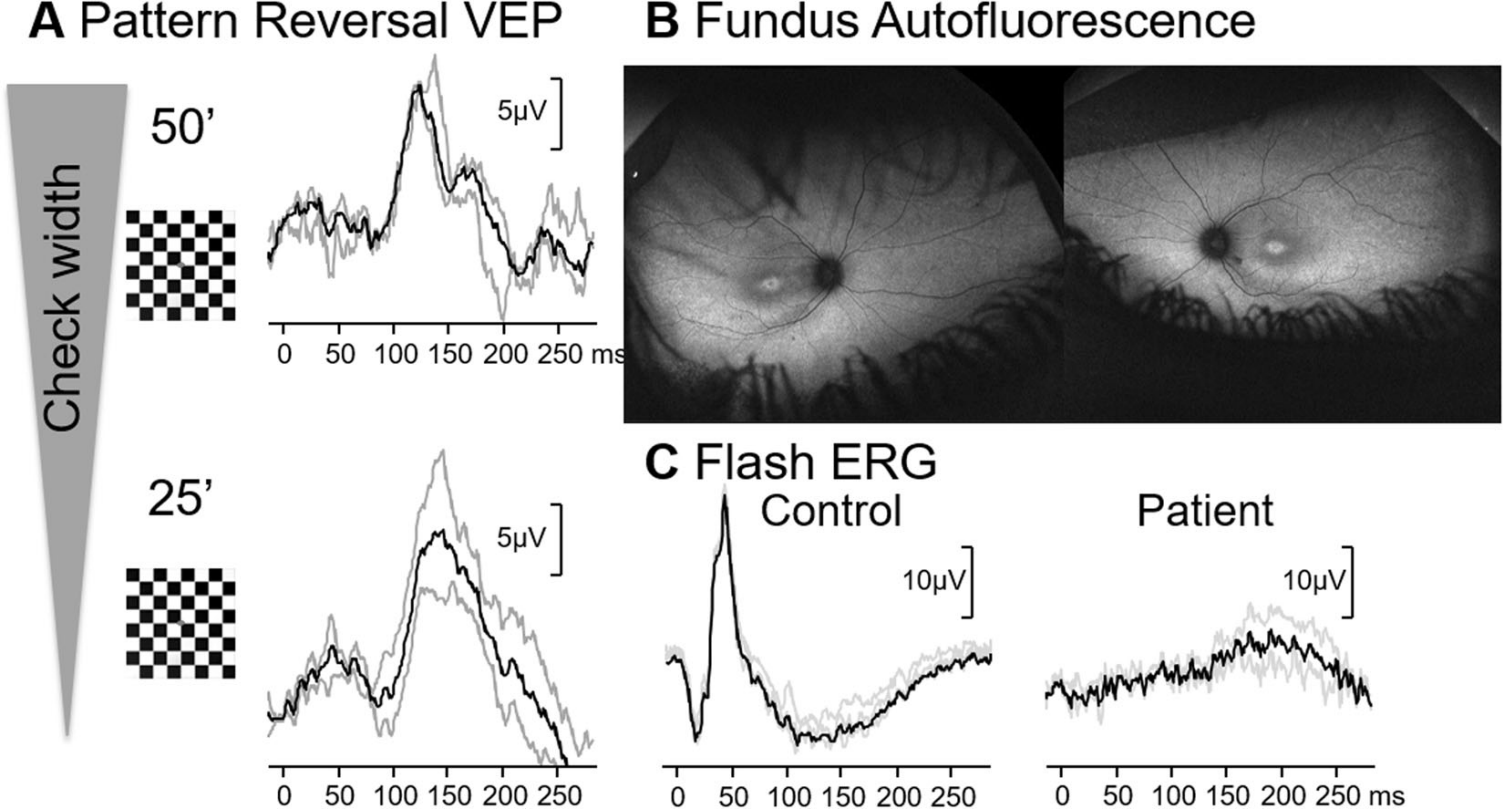
Electrophysiological and imaging findings from case 3 – a girl with Bardet-Biedl syndrome. Pattern-reversal VEP to two different check size (**A**), widefield autofluorescence imaging (**B**) and flash ERG (**C**) are shown.

A 4-year-old girl presented for ophthalmology assessment due to developmental concerns, clumsiness and excessive weight gain. The patient had been under local ophthalmology care since infancy for esotropia and suspected strabismic amblyopia. Her visual acuity at presentation was RE 0.5 and LE 0.2 using Crowded Kays (LogMAR). VF to confrontation were attempted but were inconclusive. Visual electrophysiology was carried out (Fig.  3a–c). Pattern-reversal VEP were tested to check widths of 50’ (ISCEV large) and 25’. Responses were within reference ranges and of good morphology, suggesting good macular pathway function of either eye. Flash ERGs were carried out to a range of photopic and scotopic stimuli, and no retinal responses were evident to any of the stimuli. Fundus autofluorescence imaging was also carried out, (Fig. 3b), which showed a central area of hyper autofluorescence, surrounded by a ring of relative hypo autofluorescence. While conclusive VF testing has never been possible in this child, we can take from the electrodiagnostics that there is severe dysfunction of the peripheral retina (absent skin ERGs) and relative preservation of the macula as it can support reasonably good pattern-reversal VEPs. Therefore, in this clinical picture, VF constriction with macular sparing can be assumed. However, an absent ERG can not provide a quantitative measure of the degree of VF constriction. The child was later diagnosed with Bardet-Biedl syndrome, explaining the ciliopathy associated retinal dystrophy.

### Case 4: Constricted visual fields due to malingering

**Fig. 4:**
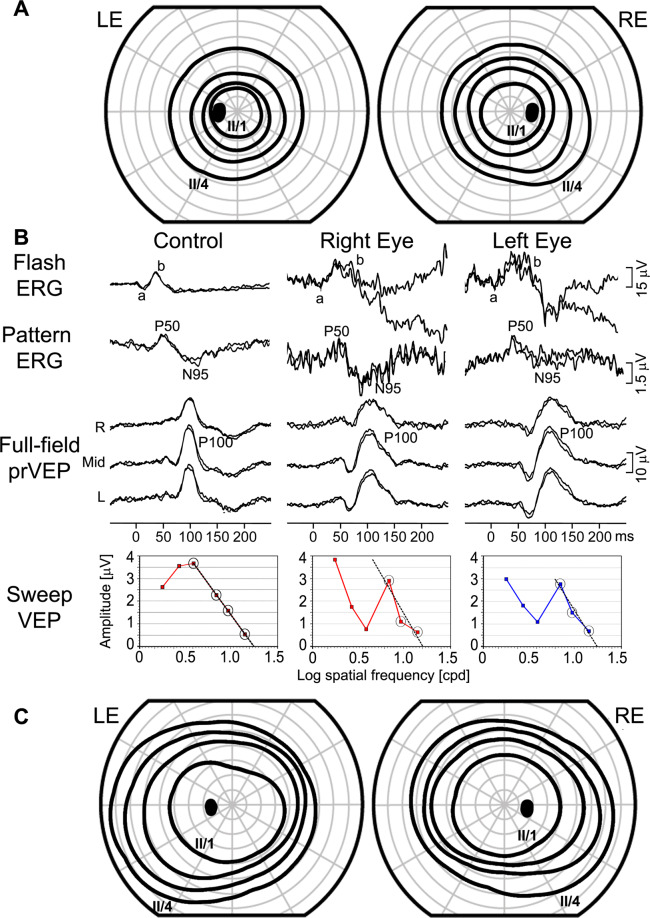
Electrophysiological and visual-field findings from case 4: a girl complaining of progressive vision loss. Campus Goldmann visual-field testing at presentation showing constriction (**A**). Normal electrophysiological findings (**B**). Note the artefacts during flash and pattern ERG caused by excessive blinking. R—right occiput, Mid—mid-occiput, L—left occiput. The main components of the flash ERG (a and b wave), pattern ERG (P50 and N95) and pattern-reversal VEP (P100) are labelled. Repeated visual-field testing (within 2 days) showed normalisation confirming malingering (**C**). Visual fields were performed with II/1 – II/4 stimuli.

An 11-year-old girl was referred for further investigation due to complaining of progressive vision loss. The girl had a 5 months history of progressive deterioration of vision in both eyes, her visual acuity had gradually decreased from 0.1 to 0.7 (LogMAR) in both eyes, with bilateral visual-field constriction (Fig.  4a). Ophthalmological and neurological investigations including MRI were all normal. Electrophysiology testing was undertaken (Fig. 4b), with poor co-operation during the ERG recording, and she did not tolerate skin electrodes. Never the less, although noisy from artefacts both the flash and pattern ERG were recordable. The pattern-reversal VEP was normal and sweep VEP predicted normal visual acuity. The electrophysiological findings therefore did not reveal any functional deviations that would explain low visual acuity and constricted visual fields. All clinical tests were carefully repeated the next day and were within normal limits including excellent visual acuity and normal visual fields (Fig. 4c).

## Central visual-field defect

Central VF defect or central scotoma can appear either as a uni- or bilateral field defect and has a long list of possible causes. It can result from a lesion in the papillomacular bundle of retinal ganglion cell axons, either because of choroidal, outer or inner retinal disorder in the macular region or as a consequence of optic nerve lesion. It is usually accompanied by a decrease of visual acuity, spatial contrast sensitivity and colour perception [23]. The eye conditions, commonly associated with central visual-field defect are macular disorders such as; macular dystrophies, age-related macular degeneration, retinal haemorrhage, macular oedema, macular hole, central serous chorioretinopathy, cone dystrophy, cone-rod dystrophy and optic neuropathies such as optic neuritis, or primary ganglion cell disease such as Leber hereditary optic neuropathy and dominant optic neuropathy [58]. If scotoma includes both the central region and the physiologic blind spot, it is described as centrocecal scotoma and might be associated with many types of optic neuropathies [23].

Central scotoma can be easily detected with electroretinographic testing, as both the PERG, and the pattern-reversal VEP reflect activity arising from the central visual field [59]. The pattern ERG provides an objective measure of central retinal function [60] but is dependent on the stimulus field size [39]. Therefore, with a larger stimulus field size also, non-macular regions contribute to the signal [59]. On the contrary, the pattern-reversal VEP is dominated by the macular region [59] and reflects the activity of cortical neurons representing the central 6–12° of the visual field [38], with most of the signal arising from central 4 [61]. Thus, an abnormal pattern-reversal VEP has been shown in control subjects to be a sensitive indicator of a central scotoma [62–64]. The pattern ERG has two components, the P50 and the N95, that can be used to further distinguish the level of dysfunction causing the central field loss. Both the P50 and the N95 reflect macular retinal ganglion cell function and depend on the functional integrity of the macular cones [60]. However, there is a considerable contribution from more distal retinal elements to the P50 component only [65]. An abnormality of P50 indicates distal macular dysfunction, while the preservation of P50 and selective abnormality of N95, accompanied with abnormal pattern-reversal VEP indicates dysfunction at the level of the retinal ganglion cells and optic nerve [16, 60]. Therefore, in case of central scotoma, simultaneous recording of PERG and pattern-reversal VEP is used to distinguish optic nerve dysfunction from macular disease, especially when there is no other clear clinical abnormality [16]. This principle of simultaneously recording the PERG and pattern-reversal VEP is used frequently throughout visual electrophysiology testing because of the increased diagnostic yield (see also bi-temporal hemianopia). With additional use of ffERG testing further evaluation of retinal abnormality is possible as well as differentiation between maculopathy and generalised cone or cone-rod dystrophy [14, 60].

It is possible to reliably record the PERG as well as the ffERG with skin electrodes (rather than corneal electrodes), which are well tolerated even with infants and small children [66, 67]. The recording of the PERG and ffERG using skin rather than corneal electrodes results in a smaller amplitude signal recorded. The recording of a PERG using skin electrodes is not universal practice as the recording of the amplitude is so small it may not be possible to record this above the levels of background noise, which is not an indication of dysfunction. See case 4 as an example of how small this signal is compared to the flash ERG, the P50 component of the skin PERG recorded in this case is less than 2μV. The mfERG has better spatial resolution than the PERG and full-field ERGs, and enables further characterisation of the size and location of macular abnormality, but requires steady fixation, reliable co-operation, as well as the use of contact electrodes [16] making it difficult for paediatric patients. One study has shown that the mfERG can be reliably recorded in healthy full-term children from 5 years of age and above with the use of topical anaesthesia to aid tolerance to corneal electrodes [68]. The mfERG was used to establish an early diagnosis of macular dysfunction in two paediatric cases with Stargardt disease using topical anaesthesia [69]. Stargardt disease is one of the most common inherited macular dystrophies, presented with bilateral central visual loss, central scotoma and characteristic appearance at the posterior pole. Onset is most common in childhood or early adulthood [70]. Stargardt disease can be classified into three subtypes, according to the level of electrophysiological abnormality. Group 1 represents an isolated macular dysfunction (abnormal PERG/mfERG, normal ffERG), Group 2 has additional generalised loss of cone function (abnormal PERG/mfERG and LA responses of the ffERG) and Group 3 has a generalised loss of both cone and rod function (abnormal PERG/mfERG and ffERG) [71, 72]. These three subtypes show differences in progression of the disease, with Group 1 being associated with the best and Group 3 with the worst prognosis [72]. In children diagnosed with Stargardt disease, electrophysiological assessment is therefore valuable, not only to evaluate the extent of retinal abnormality, but also to provide early information on prognosis.

Lesions of the optic disc or axial lesions of the optic nerve may involve sufficient papillomacular axons to cause a central or centrocecal scotomas [23]. Optic neuritis is one of the possible causes of such scotomas in children. Loss of VF is described in the acute stage of the disease, followed by a rapid recovery and complete resolution after 6 months [73]. The pattern-reversal VEP is usually delayed and reduced in amplitude during the acute stage, but it can recover over the time [14, 74]. One study of 101 patients with optic neuritis found that at first episode of optic neuritis 50% were identified with OCT alone and 67% with VEP alone. However, when both techniques were combined this rose to 75% significantly improving the sensitivity than either test in isolation and rising again to 95% with repeated episodes [75]. The prognosis of childhood optic neuritis is generally better than in adults and studies have shown a 42%–55% incidence of VEP normalisation at follow-up [73, 76]. However, retrograde degeneration of the retinal ganglion cells can occur, seen as a selective PERG N95 reduction and reduced pattern-reversal VEP amplitude. Such electrophysiological findings indicate irreversible axonal loss, lack of visual improvement and poor prognosis [14, 74]. A prominent N95 reduction in the PERG that occurs already in the acute stage of disease, might help to distinguish primary ganglion cell dysfunction, such as Leber hereditary optic neuropathy [14, 77, 78] from other types of optic neuropathies that can also present in childhood with acute painless visual loss and central scotoma [78, 79].

Compressive lesions of the optic nerve, such as optic nerve glioma, might be another reason for visual loss in children [14]. Although patients with optic pathway gliomas can demonstrate various types of field defects, from generalised depression to hemianopias, about 70% demonstrate a central scotoma or central depression on visual-field testing [80]. Formal visual-field testing is problematic in young children and optic pathway glioma is associated with additional intellectual disability. In a study of 40 children with optic pathway gliomas only 15 of the older ones were able to do perimetry [81]. For this reason VEPs are often used clinically to provide an additional objective method for monitoring of these patients detecting visual dysfunction in 68% of children with low-grade glioma [82]. In the reported 15 children with optic pathway glioma who could undertake both perimetry and visual-evoked potentials VEP sensitivity to a field defect was reported to be between 0.94 and 1.0 (specificity 0.33–1.0) [81]. In a study of 26 adult NF1 participants pattern VEPs are shown to have a significant difference in amplitude (*p* < 0.001) and latency (*p* < 0.001 to p0.005 with change in check width) compared to controls [83]. In the same adult group the sensitivity of the frequency doubling technology perimetry was between p0.0004 and <0.001 depending on measure used.

The following two cases reveal typical electrophysiological findings in paediatric patients with central scotoma, one associated with retinal disease and the other with optic nerve disease.

### CASE 5: Central scotoma caused by Stargardt disease

**Fig. 5:**
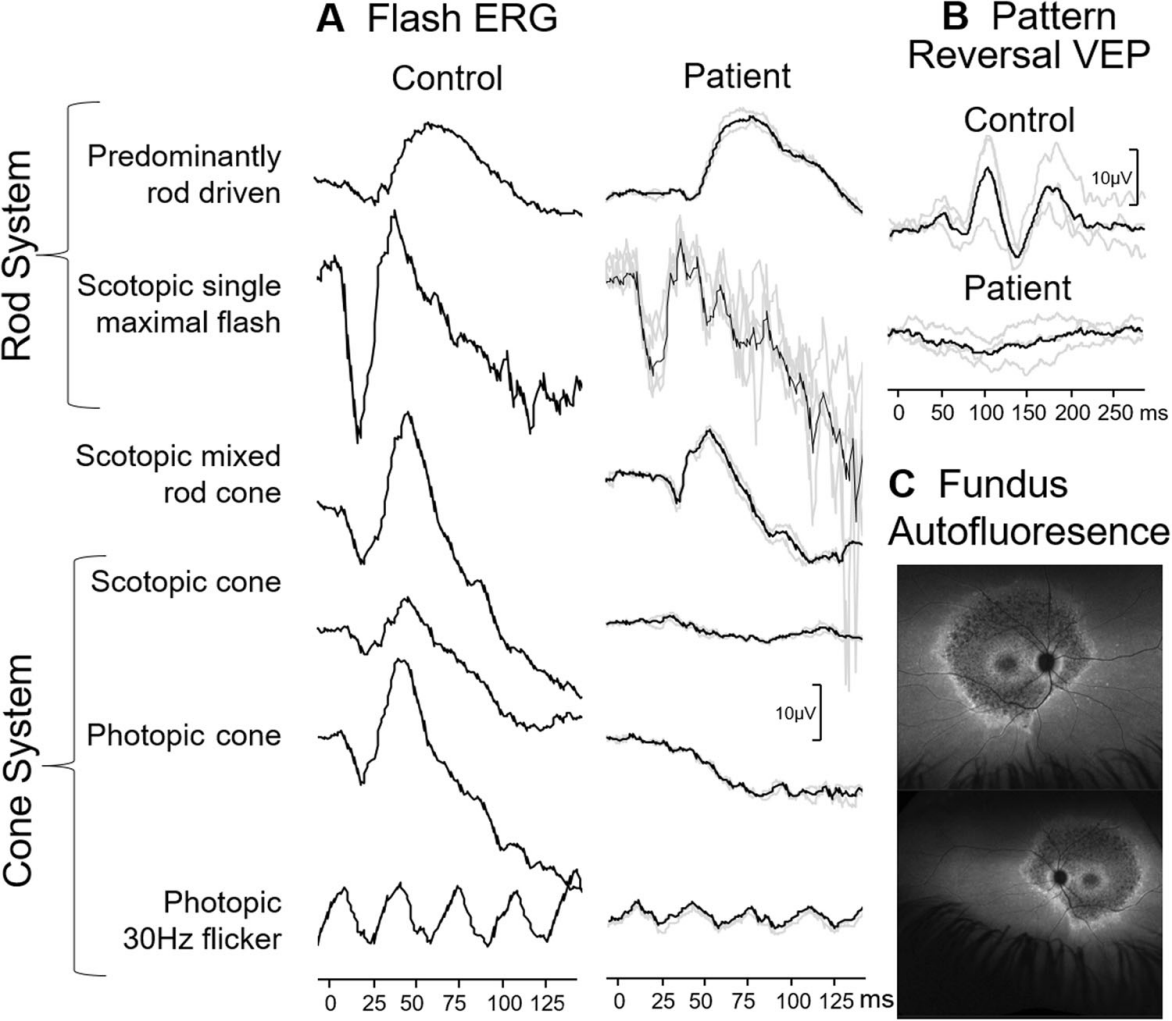
Electrophysiological and imaging findings from case 5: a girl diagnosed with Stargardts disease. Flash ERG (**A**) carried out to a range of rod and cone system stimuli. The pattern-reversal VEP (**B**) was absent. OPTOS fundus auto-fluoresence images showing the extent of the macular involvement for each eye are shown (**C**).

A 6-year-old female presented with a vague history of deteriorating reading skills. Her visual acuity was RE 1.3 and LE 1.0 (LogMAR), with no significant refractive error found. Colour vision was tested and all presented Ishihara plates were negative. Fundus appearances revealed bilateral macular atrophic changes at the level of the photoreceptors/RPE, consistent with a cone or macular dystrophy. Electrodiagnostic tests were carried out (Fig.  5), which identified that she had cone responses to flash stimulation within the noise level, while her 30 Hz flicker responses were still evident, but reduced in amplitude. She had normal rod system ERG responses in either eye. She was not able to cooperate for a PERG recording, while her pattern-reversal VEP was very abnormal, indicating loss of macular function. Genetic testing was later carried out confirming a mutation in the ABCA4 gene and a diagnosis of Stargardt disease.

### CASE 6: Central scotoma caused by Optic neuritis

**Fig. 6:**
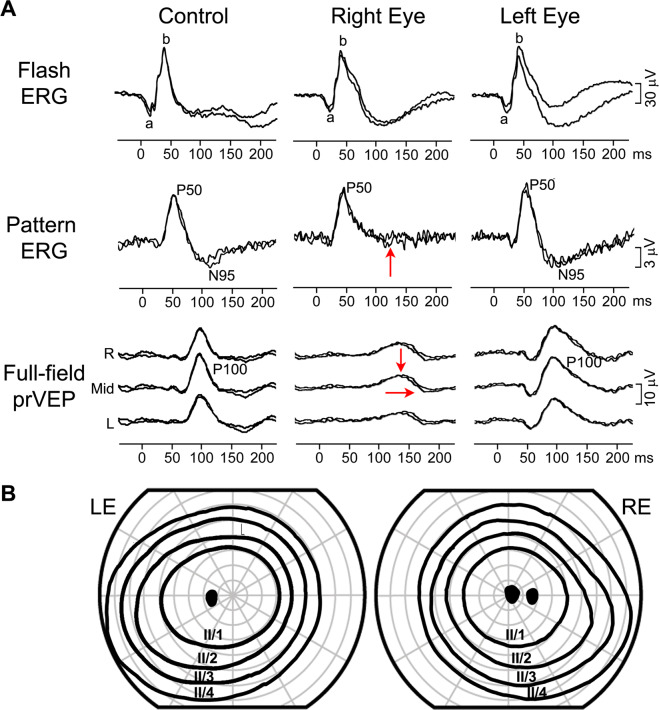
Electrophysiological and visual-field findings from case 6: a boy with central scotoma on the right eye due to optic neuropathy. Electrophysiology testing (**A**) showed a decrease of N95 wave of the pattern ERG and a decrease and prolongation of the VEP wave P100 (both indicated with red arrows) for the right eye. R— right occiput, Mid—mid-occiput, L—left occiput. The main components of the flash ERG (a and b wave), pattern ERG (P50 and N95) and pattern-reversal VEP (P100) are labelled. (**B**) Campus Goldmann VF testing revealed central scotoma on the RE. Visual fields were performed with II/1–II/4 stimuli.

A 16-year-old boy presented with a recurrence of RE optic neuritis with RE visual acuity reduced to 0.8 (LogMAR). At the first episode 4 years ago RE vision was reduced to counting fingers at 0.5 metres but recovered to normal 3 months later. Ophthalmological examination also revealed temporal pallor of the right optic disc and profoundly affected colour vision of his RE, as well as a central scotoma in the visual field of the RE. Electrophysiological findings and VF are summarised in Fig.  6 and showed for his affected eye normal flash ERG and normal P50 of the pattern ERG, while there was a marked reduction of the N95, and abnormality of the pattern-reversal VEP P100, which was reduced and significantly prolonged. In comparison with the electrophysiological results recorded 4 years earlier (3 months after the first episode of optic neuritis once it had recovered and visual acuity was normal), there was an additional decrease of the PERG N95 and VEP amplitude, indicating that another episode of optic neuritis occurred in the meantime, leading into additional retrograde degeneration of the ganglion cells and irreversible atrophy of the optic nerve fibres.

## Bi-temporal hemianopia

Paediatric brain tumours of the suprasellar region, although varied in pathology, share the same proximity to the optic chiasm where they can invade or compress causing vision loss [84]. As well as tumours such as craniopharyngioma, glioma, pituitary adenoma and germ cell tumours compressive cysts can also form in the sellar/suprasellar region in childhood, including arachnoid and Rathke Cleft Cysts. Any of these compressive lesions can result in a bi-temporal hemianopia if the chiasm is compressed.

In dense bi-temporal hemianopia there is a functional deficit of crossing fibres at the chiasm and electrophysiologically a “crossed asymmetry” is observed. This trans-occipital asymmetry of VEP distribution is called a “crossed asymmetry” if the VEP asymmetry seen switches occipital sides for each eye stimulated, e.g., larger on the right occiput for the RE, but larger on the left occiput for LE [85]. A “crossed asymmetry” is also seen when there is an overcrossing of fibres at the chiasm as in human albinism [86, 87]. This distribution is different to that described in “uncrossed asymmetry”, which is described in the section homonymous hemianopia.

**Fig. 7:**
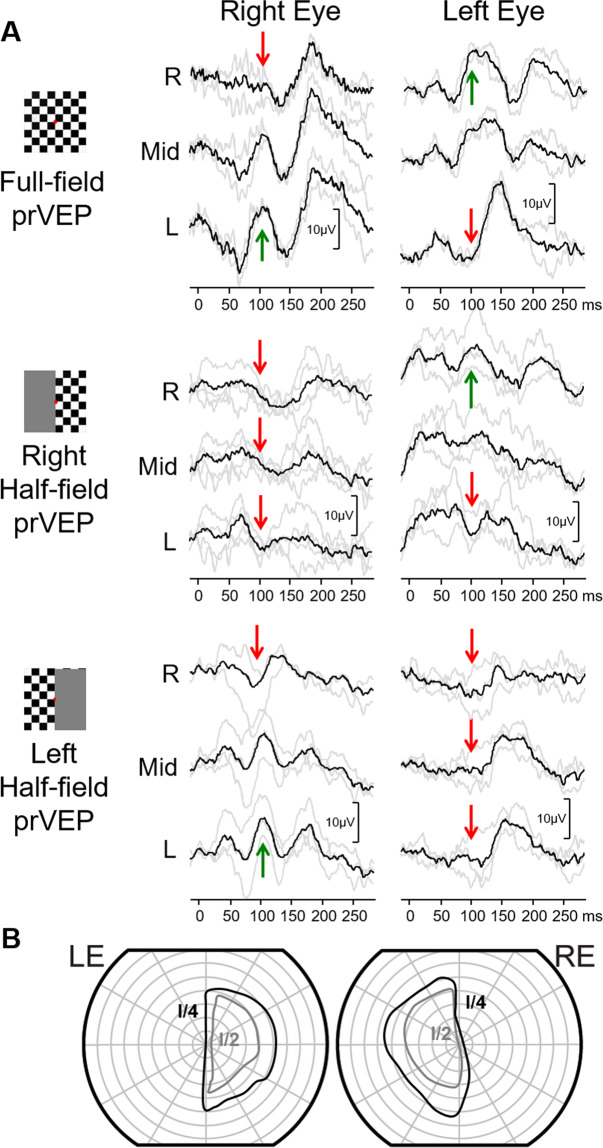
Electrophysiological and visual-field findings from case 7: a 4-year old girl with a chiasmal tumour. Pattern-reversal VEPs (**A**) from the right and left eye to full field, right and left half-field stimulation. R—right occiput, M—mid-occiput, L—left occiput. Visual fields at age 7 from the patient showing the bi-temporal hemianopia tested with I4e and I2e stimuli (**B**).

Pattern-reversal VEP produced to a large field stimuli and using a mid-frontal reference are known to, paradoxically, lateralise [88, 89]. This means they are detected over the opposite side of the head to the hemisphere they come from (i.e., a response from the right hemisphere is detected over the left occiput). This has been shown to be a result of the medial/oblique orientation of V1 fibres, projecting the VEPs towards the electrodes over laying the opposite hemisphere [90]. For a schematic of paradoxical vs normal lateralisation, see Marmoy et al. [91]. In a crossed asymmetry associated with a functional deficit of crossing fibres as in bi-temporal hemianopia the following distribution is observed as seen in case 7, (Fig. 7). During full-field pattern-reversal stimulation of the RE the main positivity is seen over the left occiput and in contrast during LE stimulation the main positivity is seen over the right occiput. This mirror image in distribution across the back of the head, with the main positivity flipping sides of the head as each of the two eyes is tested is called a crossed asymmetry. Multichannel data can be analysed using a Pearson’s correlation coefficient to quantify the extent of the crossed asymmetry. This has been shown to be more accurate (sensitivity 86% and specificity 81%) than visual inspection of the waveforms (71% sensitivity 81% specificity) in the crossed asymmetry associated with albinism [92]. While the crossed asymmetry of albinism is associated with overcrossing at the chiasm this technique can also be used in all crossed asymmetries, even where there is undercrossing at the chiasm as illustrated in cases of achiasmia [74, 93].

Half-field stimulation was carried out to investigate this further, where the pattern-reversal stimuli is presented on each the right- and left-half of the screen individually to test each the right and left half-field individually. Note how in case 7 when the functional half-field of either eye is stimulated the distribution is almost identical to that seen during full-field stimulation. When the non-functional half-fields of either eye are stimulated no responses are evident.

Compressive chiasmal abnormalities have been reported to be detectable in 29–100% (Table  1) of cases using only monocular full-field VEPs and a range of recording parameters [94–96]. As with formal perimetry, monocular testing is essential to the assessment of a suspected chiasmal problem, resulting in a bi-temporal hemianopia. Monocular VEPs can be challenging to test in children, but have been reported repeatedly to be useful in detecting crossed asymmetries in children with chiasmal tumours unable to perform formal perimetry [74]. Using half-field VEPs in addition to full-field VEP has been shown to improve detection of crossed asymmetry [94–98] (Table 1).

**Table 1:** A summary of the reported sensitivity rates of pattern-reversal visual-evoked potentials (prVEPs) in different chiasmal and post chiasmal visual-field defects.

Location	Visual-field loss	VEP stimuli	Reference	*n*	Stimuli details	Reported VEP sensitivity	Reported VEP sensitivity mean
Chiasm	Bi-temporal hemianopia	Full-field prVEP	[94]^a^+[85] Halliday et al.	8	50’ prVEP	100%	79.3%/81%
			[94]^a^ Holder	10	13’ and 26’ prVEP	100%	
			[94]^a^ Gott et al.	12	50’ prVEP	100%	
			[94]^a^ Onofrj et al.	3	13’ prVEP	100%	
			[94]^a^ Holder and Bullock	34	13’ and 26’ prVEP	100%	
			[94]^a^ Vitova et al.	13	prVEP	92%	
			[94]^a^+[97] Brecelj	50	50’ prVEP	90%	
			[94]^a^ Camacho et al.	9	58’ prVEP	89%	
			[94]^a^ Muller-Jensen et al.	27	46’ prVEP	85%	
			[95] Brecelj et al.	38	50’ prVEP	66%	
			[94]^a^+[101] Flanagan and Harding	9	56’ prVEP	55%	
			[94]^a^ Stark and Lenton	13	20’ and 40’ prVEP	54%	
			[94]^a^+[96] ﻿Haimovic and Pedley	15	31’ prVEP	29%/53%	
			[94]^a^+[117] Maitland et al.	8	50’ prVEP	50%	
		Half-field prVEP	[94]^a^+[96] Haimovic and Pedley	15	31’ prVEP	73%/100%	85.5%/92.25%
			[94]^a^+[97] Brecelj	50	50’ prVEP	100%	
			[94]^a^+[101] Flanagan and Harding	9	56’ prVEP	89%	
			[95] Brecelj et al.	38	50’ prVEP	80%	
Post chiasmal pathway	Quadrantanopia	Full-field prVEP	[116] Blumhardt et al.	8	50’ prVEP	37%	
		Half-field prVEP	[118] Celesia et al.	5	1°4’ prVEP	40%	20%
			[96] Haimovic and Pedley	2	31’ prVEP	0%	
		Quadrant VEP	[115] Báez Martín et al.	24	16’ prVEP	100%	87.5%
			[121] Bradnam et al.	2	90’ prVEP	75%	
	Homonymous Hemianopia	Full-field prVEP	[116] Blumhardt et al.	15	50’ prVEP	54%/ 65%	44%/47.6%
			[128] Kuroiwa and Celesia	16	40’ prVEP +tVEP	50%	
			[96] Haimovic and Pedley	7	31’ prVEP	28%	
		Half-field prVEP	[96] Haimovic and Pedley	12	31’ prVEP	100%	90.26%/91.06%
			[128] Kuroiwa and Celesia	16	40’ prVEP + tVEP	94%	
			[116] Blumhardt et al.	15	50’ prVEP	92%	
			[118] Celesia et al.	29	1°4’ prVEP	82/86%	
			[117] Maitland et al.	12	50’ prVEP	83.3%	

In one paper a transient VEP (tVEP) was also used [128]. Note how the sensitivity to full-field stimuli reduces further back from the chiasm, and the use of more complex stimuli (half or quarter fields) improves sensitivity. The number of participants in the study is shown (*n*).

^a^Is a review paper that has extracted data on full-field pattern VEPs from 13 papers and half-field VEPs from three papers. Where the authors have explored more than one criteria for diagnosing a VEP deficit or different classifications of the visual-field defect both values have been recorded. The mean has then calculated twice to reflect these different values.

In a case series of 10 children with craniopharyngioma, visual fields were not possible in the two youngest children (aged under 7) yet OCT was possible in all [7, 99]. Good correlation has been shown between VF and OCT in children with craniopharyngioma [7, 99] yet as previously discussed the time course to see these changes on OCT remains a limitation of this technique [11]. In 21 children presenting with optic pathway glioma visual acuity was normal in both eyes of 4 of the children, and in one eye of 7 of the children, yet all of these had an abnormal VEP [100].

**Fig. 8:**
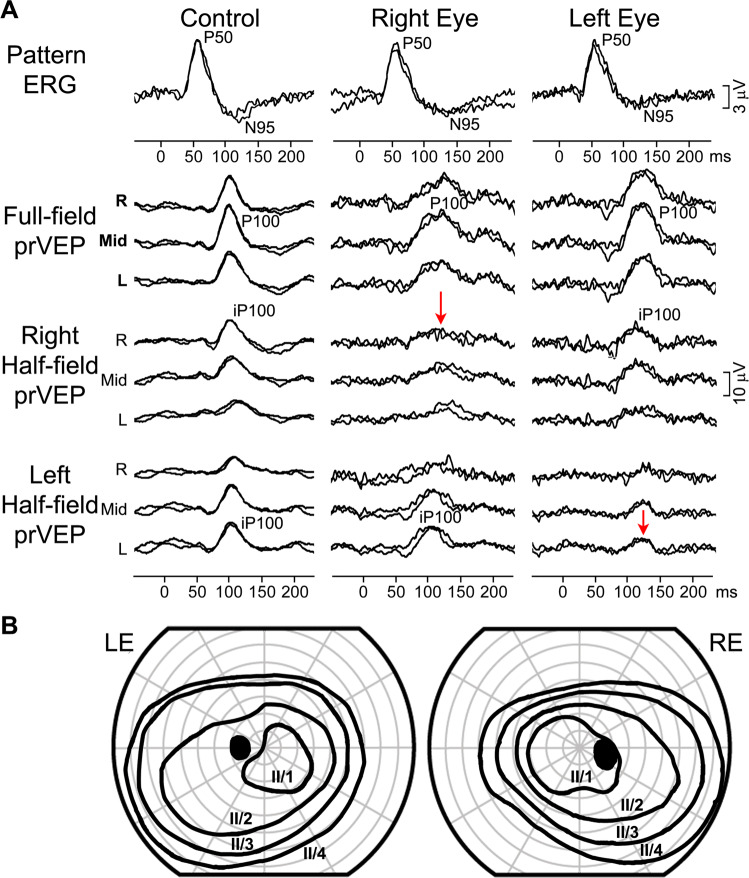
Electrophysiological and visual-field findings from case 8: -a girl with tumour formation in the sellar area. **A** Electrophysiological findings. Normal VEP presentation shows equal distribution of the response over the right (R) and left (L) lateral occipital electrode with full-field stimulation, higher amplitude over the right electrode with right half-field stimulation, while with the left half-field stimulation, the amplitude is higher over the left electrode. There was a relative decrease of the VEP amplitude (indicated with red arrows) over the two lateral electrodes with the stimuli that presented a temporal half-field for each eye (right half-field for the RE and left half-field for the LE). R —right occiput, Mid—mid-occiput, L—left occiput. The main components of the pattern ERG (P50 and N95) and the ipsilateral (i) pattern-reversal VEP (P100) are labelled. **B** Visual-field testing revealed bi-temporal hemianopsia when using the smallest light stimulus. Visual fields were performed with II/1–II/4 stimuli.

Kelly and Weiss [81] reported analysis of the trans-occipital asymmetries in the VEPs of their patients with chiasmal optic pathway glioma did not detect hemianopia in a third of patients. They pointed out that in the patients where hemianopia was not detected there was a small signal to noise ratio, meaning that the responses recorded were very small across all of the electrodes making interpretation difficult. This is logical in optic pathway glioma where the lesion may result in interruption to both the crossed and non-crossed fibres at the chiasm and/or the whole of one or both optic nerves reducing the amplitude of the signal recorded. If the amplitude of the VEPs recorded is small across all channels it makes the interpretation of any trans-occipital asymmetry more difficult. In contrast, there have been many reported cases of progressive unilateral optic nerve lesions where VEPs have detected early chiasmal involvement of the lesion, at times before this is detectable on perimetry [74, 85, 94, 95, 101]. This is similar to that presented in case 8 (Fig. 8) where only a subtle bi-temporal defect is evident on VF testing despite the marked trans-occipital VEP asymmetry. In the monitoring of such paediatric patients VF testing where possible is complemented with electrophysiology.

As discussed in central scotoma, simultaneous recording of the pattern-reversal VEP and the PERG has been shown to add useful information by testing retinal ganglion cell function [94, 102, 103]. As previously discussed, this usually requires the use of corneal electrodes and is not tolerable to all children. However, some units have managed to record these using skin electrodes, this is technically challenging due to the small signal detectable and not universal practice [103] only possible in rare cases. An example of how small a PERG response recorded with skin electrodes is, is shown in case 4 (Fig. 4).

### Case 7: Bi-temporal hemianopia from a chiasmal tumour with typical VEP findings

A 4-year-old girl presented for an ophthalmology review after recent diagnosis of NF1 related optic pathway glioma. The primary glioma was situated in the optic chiasm affecting the left side, slightly more than the right. Her electrophysiology showed the pattern of crossed asymmetry in keeping with a relative bi-temporal hemianopia (Fig. 7). This has remained stable over 3 years of follow up, and at age seven, visual fields were possible confirming the bi-temporal hemianopia.

### Case 8: Chiasmal lesion with atypical bi-temporal hemianopia

A 12-year-old girl presented for an ophthalmology review after an episode of severe headache and bilateral optic nerve oedema. An MRI scan detected a large Rathke cyst of the pituitary gland. At her follow-up visit OCT demonstrated reduced optic disc oedema bilaterally, visual acuity was normal, and her colour vision had improved. Electrophysiological examinations were performed several times over the 6 months observation period. The PERG was normal from both eyes, indicating preserved ganglion cell function of either eye (Fig. 8A). The full-field pattern-reversal VEPs (Fig. 8A) indicated macular pathway dysfunction affecting the right eye more than the left, potentially associated with the bilateral optic nerve oedema. The VEP abnormality was most clearly seen, when half-field stimulation was applied, the P100 was decreased on the RE with right half-field stimulation, and relatively decreased on the LE with the left half-field stimulation. The reduced pattern-reversal VEP response from each eyes with temporal half-field stimulation is a crossed asymmetry pattern. This indicates an element of chiasm dysfunction and a suggests a relative bi-temporal deficit. Her visual field showed a relative bi-temporal defect only when testing with the smallest light stimulus (Fig. 8B).

## Altitudinal visual-field defect

A true altitudinal VF loss is characterised by loss of all or part of the superior or inferior half of the visual field. The loss does not cross the horizontal median. Common causes in adults include ischaemic optic neuropathy, hemibranch retinal artery occlusion, retinal detachment, advanced glaucomatous optic neuropathy, optic nerve or chiasmal lesion and optic nerve coloboma. Most common altitudinal VF defect is due to ischaemic reasons, but since these are very rare in paediatric population, they will not be extensively discussed here, though visual electrodiagnostic methods are diagnostically and prognostically useful in these cases [104]. In the paediatric population dorsal stream dysfunction as part of the spectrum of cerebral visual impairment (CVI) can cause inferior visual-field loss with specific symptoms related to development age/ability. Periventricular leukomalacia is often a consequence of white matter damage in prematurity and a cause of lower visual-field defects [105]. These visual-field defects are usually described as being inferiorly constricted and often do not strictly follow the horizontal median [106]. The inferior visual field is important in children for navigating down stairs and reading.

**Fig. 9:**
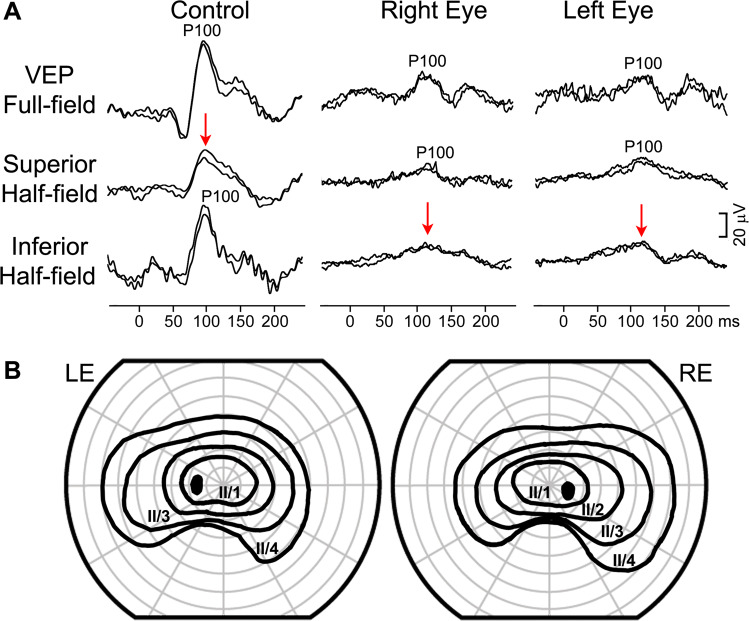
Electrophysiological and visual field findings from case 9: a child with periventricular leukomalacia. **A** In controls the VEP is of larger amplitude with inferior half-field pattern-reversal stimulation and smaller amplitude with superior half-field stimulation. In each eye of the patient the amplitudes were lower with inferior half-field stimulation (indicated with red arrows). **B** Campus Goldmann visual-field-testing revealed bilateral inferior field loss. Visual fields were performed with II/1– II/4 stimuli.

VEPs to lower and upper field stimulation have been utilised previously [107, 108]. Pattern-reversal VEPs to lower half-field stimulation show higher amplitude and shorter latency compared to upper field response in healthy adults [108]. The density of cone photoreceptors and retinal ganglion cells are significantly greater in the superior retinal quadrants [109], which suggests that the visual spatial resolution is better in the lower visual field. This was confirmed with many psychophysical tests [110]. Cortical retinotopic organisation shows that the area that represents the lower VF is more exposed to the skull and scalp, than the area that represents the upper VF [89]. These VEP characteristics can be utilised both in studying upper [111] and lower VF defects [112]. However, if the VF defect does not respect the horizontal meridian and the central part is still preserved, the above described VEP asymmetry might not be evident. The majority of P100 VEP receives input from the central 6–12° of the visual field [38], so with the preservation of those parts an altitudinal field defect might be missed with VEPs to upper and lower half-field stimulation. If loss of lower VF is dense enough despite preserved central visual field, it can lead to relatively lower P100 amplitude [112]. This is because while the pattern-reversal VEP is dominated by macular contributions there are contributions from more paramacular areas. In our experience because of this challenge in interpretation, and the rarity of this clinical presentation this technique is one more rarely used in clinical practice. However, both units have used this in the investigation of children with specific CVI symptoms such as difficulty going down (but not up) stairs where inferior vision loss is suspected. As in case 9 of a girl with inferior visual-field constriction due to periventricular leukomalacia (Fig. 9).

### Case 9: VEPs in a child with inferior field loss

A 14-year-old girl with Asperger’s syndrome was routinely reviewed. Her mother described her as a clumsy child, often falling over obstacles on the floor. She was born at term, without any perinatal problems. Owing to suspicious optic discs appearance, which looked elevated, an MRI scan of the brain was done. This confirmed periventricular leukomalacia (PVL). VF testing was undertaken that revealed bilateral lower half-field defects. Her visual acuity was normal bilaterally and so was her colour vision. Pattern-reversal VEPs were tested using upper and lower half-field stimulation (Fig. 9). These showed a better defined VEP with upper half-field stimulation, on the contrary to the healthy population, where a larger amplitude VEP is usually recorded by stimulating the lower half-field.

## Quadrantanopia

Both inferior and superior homonymous quadrantanopias are most likely caused by damage to the occipital lobe, followed by temporal lobe and, least likely, parietal lobe [113]. Developmental abnormalities of the temporal lobe of children can be caused by a diverse range of pathology [114]. These abnormalities can lead to intractable temporal lobe epilepsy requiring temporal lobectomy. Children who undergo temporal lobe surgery are at risk of superior quadrantanopia because of the protrusion of Meyers loop through the anterior temporal lobe [115].

Full and half-field pattern VEPs are known to be insensitive to picking up well-defined superior or inferior quadrantanopia in 0-40% [96, 116–118] (Table 1). However, the pattern-reversal stimuli can be divided further to individually test the four quadrants increasing sensitivity [115, 119–121] (Table 1). However, such stimuli might also fail to detect the abnormality where the upper quadrantic defects are incomplete, as shown in these two published cases [122]. Both cases showed symmetrical amplitude responses within the normal range, with 20’ stimulation, while a relative amplitude reduction from the affected quadrant after 50’ stimulation was detected in one patient only [122].

**Fig. 10:**
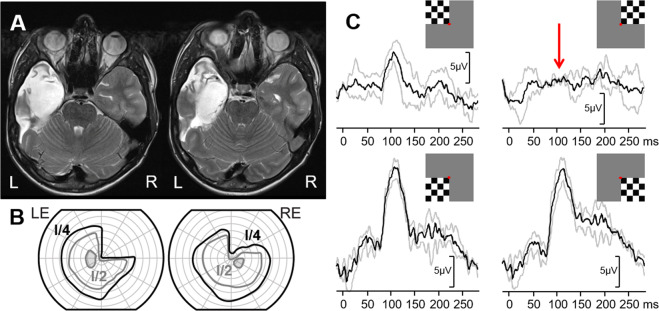
Electrophysiological, imaging and visual-field finding from case 10: a child who had undergone a temporal lobectomy. **A** MRI scans of the patient showing the affected left temporal lobe, **B** visual fields showing homonymous superior quadrantanopia, **C** pattern-reversal VEPs tested to four quadrants. Note the absence of response to superior right stimulation in keeping with the visual field. Note the larger amplitude to inferior stimulation than the unaffected superior left stimulation. The inferior right and left quadrant responses are very comparable.

As discussed in altitudinal field defects, the lower visual-field pattern-reversal VEP response shows higher amplitude and shorter latency compared to the upper field response [89, 108] this can be observed with superior and inferior left quadrant stimulation in case 10 (Fig. 10). This means that the main positivity to each of the inferior quadrants can be a useful comparator to each other (note how similar inferior right and inferior left quadrants are in Fig.  10), and the main positivity to each of the superior quadrants can be compared to each other (note in Fig. 10 how different superior left and superior right quadrants are).

As there are four quadrants to test, this takes twice as much time as testing with half fields. However, in children with proven robust binocularity and a suspected homonymous defect, this can be undertaken with both eyes open, as in case 10 (Fig. 10) rather than as a monocular test, which halves the amount of fixation time required. The pattern stimuli are interwoven with a cartoon of the child’s choosing and so they can have breaks to watch the TV in between each quadrant being tested. In our experience the amount of co-operation needed for a child to do quadrant testing is similar to that needed to undertake a formal visual-field test, which reduces the number of children where this testing may be appropriate to request. This is supported by a study that carried out both techniques in children after temporal lobectomy and reported quadrant VEPs to be equivalent to perimetry [115].

### Case 10: Quarter-field VEPs in superior quadrantanopia

A 7-year-old male was referred for visual-evoked potentials, post-operatively, to investigate if the resection had caused a post-operative quadrantanopia. He had undergone a left anterior temporal lobectomy for a low-grade dysembryoplastic neuroepithelial tumour, which had caused pharmaco-resistant focal epilepsy. He had an associated learning disability. His visual acuity was -0.1 (LogMAR) in both eyes, he was fully binocular, and he did not complain of any vision problems. VEPs were recorded first to quadrant stimulation. There was a reproducibly absent response to superior right stimulation. Visual fields were later undertaken that confirmed a superior right homonymous quadrantanopia.

## Homonymous hemianopia

Causes of homonymous hemianopia in infants and children are reported to include brain injury, infarction, haemorrhage or tumour in the retrochiasmal visual pathway beyond the first few millimetres of the optic tract [123].

The asymmetry of the VEP amplitude across each hemisphere was first related to hemianopia in the 1960’s [124] and since then has been further investigated in many studies. In normal subjects, they have a main positive component “P100” to pattern-reversal stimulation seen maximally at the midline. If this trans-occipital distribution of the VEP is not symmetrical, with the largest positivity over the same side of the occiput when each eye is tested, it is called an “uncrossed” asymmetry [85]. While an “uncrossed” asymmetry can localise to post chiasm, VEPs cannot differentiate between retrochiasmal lesions in the optic tract, optic radiation or occipital cortex [124]. To fully investigate this “uncrossed” asymmetry a range of stimuli should be used, including hemi-field stimulation if possible according to the child’s co-operation.

Hemi-field stimulation allows each half- or hemi-field to be individually stimulated to look for a response from each half-field. As a pattern-reversal stimulus with a large field is typically used, these responses also show paradoxical lateralisation [88, 125, 126]. Half-field VEPs are considered to be suspicious of dysfunction when there is a reproducible absence of the ipsilateral P100, the ipsilateral P100 of one half-field is more than two to four times smaller compared than the other the half-field, or the latency is significantly prolonged [96, 116, 118, 125, 126, 128]. Brecelj [127], found that all patients with complete homonymous hemianopia had half-field defects detected using VEPs to half-field stimulation, but this was less clearly seen when patients had macular sparing, or if the homonymous hemianopia was incomplete. Pattern-reversal VEP are a macular dominated response because of the large cortical representation of the macular compared to the smaller representation of the peripheral field, explaining why normally distributed pattern-reversal VEP have been reported in macula sparing homonymous hemianopia [118]. The sensitivity of half-field pattern VEPs in detecting homonymous hemianopia has been reported to be between 82-100% [96, 116–118, 128] (Table 1). However, some of these studies included incomplete homonymous hemianopia (including superior quadrantanopia) and a range of recording parameters. While half-field-testing significantly improves the detection of retrochiasmal lesions (Table 1), the sensitivity is below that of formal perimetry [118, 127, 128]. However, while not feasible in every child, in our clinical experience, half-field-testing is possible at an earlier age than perimetry is, and is a useful adjunct to confrontation methods. There have been many cautionary reports of patients where VEP to full-field stimulation was within normal limits and only half-field-testing has unmasked the defect [96, 116–118, 128–131]. We can speculate that if half-field-testing had been possible in case 12 (Fig. 12), this could potentially also have been the case. However, not all children are able to tolerate monocular half-field-testing. In uncrossed asymmetry where the suspicion is of dysfunction of one hemisphere both eyes open hemi-field-testing can be considered in binocular children [132] halving the required fixation time. Close monitoring of the child’s fixation, encouragement and plenty of breaks are essential to acquiring useful hemi-field responses as early as possible.

**Fig. 11:**
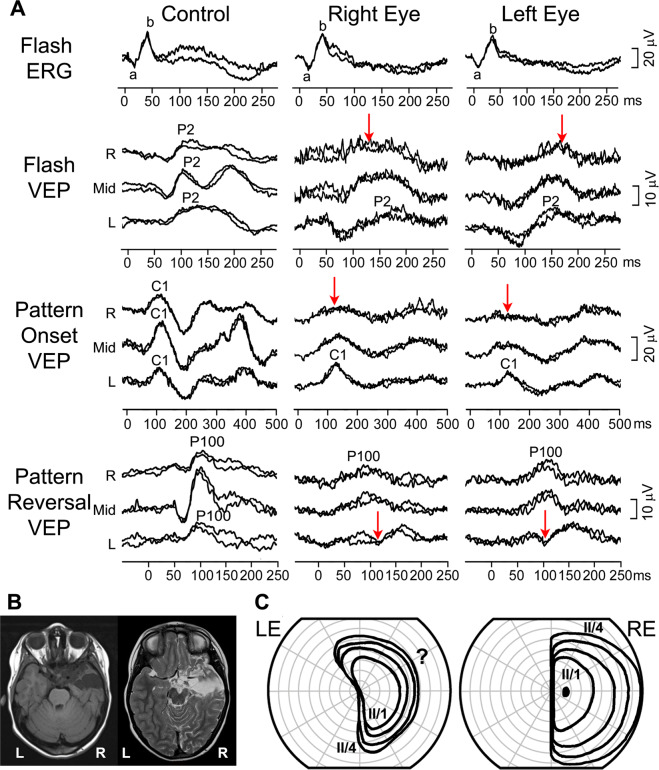
Electrophysiological, imaging and visual-field findings from case 11: a 3.5-year-old post op to a removal of a suprasellar myxoid astrocytoma. **A** In controls VEPs are overall symmetrical over the lateral two electrodes (R—right, L—left occipital electrode). In this child with left homonymous hemianopia flash and pattern-onset VEP were decreased over the right occiput (indicated with red arrows), while the pattern-reversal VEP was decreased over the left occiput. R—right occiput, Mid—mid-occiput, L—left occiput. The main components of the flash ERG (a and b wave), flash VEP (P2), pattern-onset VEP (C1) and pattern-reversal VEP (P100) are labelled. **B** MRI a few days after the operation (left) and 3 months later (right)—showing hygroma of the right temporal lobe. **C** Visual fields testing, obtained 2.5 years after the onset of visual impairment, indicating left hemianopsia. Visual fields were performed with II/1 – II/4 stimuli. ‘?’ indicates visual fields with less reliable co-operation.

As pattern-reversal and pattern-onset VEPs are known to lateralise in the opposite ways this relationship can be used to add to the VEP evidence of homonymous hemianopia [91, 133, 134]. These full-field stimuli are easier than half-field-testing for a child to comply with, making them a very useful addition. In a patient with homonymous hemianopia, if pattern-reversal VEPs were small over one hemisphere, pattern-onset VEPs should be smaller over the opposite one, as observed in case 11 (Fig. 11). More recently it has been shown that the less used offset component of the pattern-onset response lateralises paradoxically (like pattern-reversal VEPs) and so can be useful in the interpretation of these trans-occipital asymmetries [91].

**Fig. 12:**
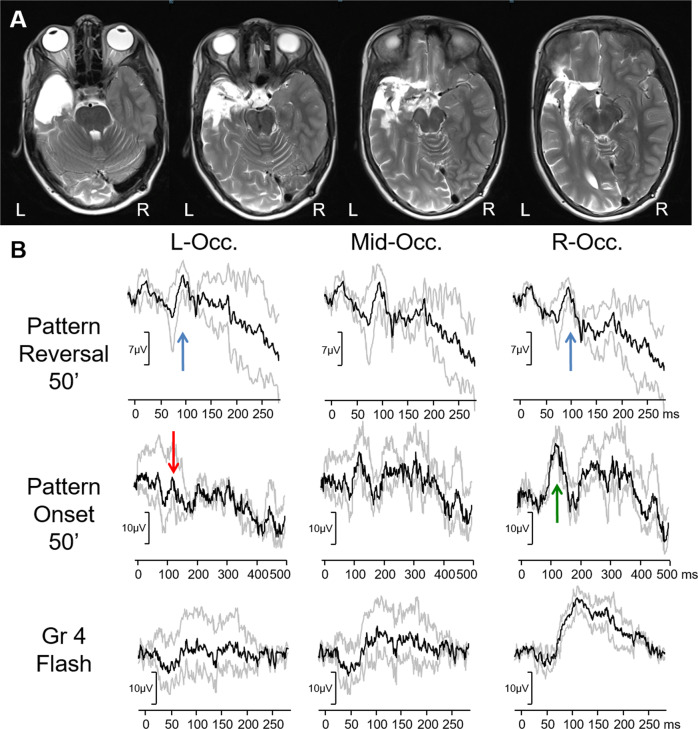
Imaging and electrophysiological findings from case 12: a 10-year-old who had a left hemispherectomy. **A** MRI slices showing the enlarged affected left hemisphere that has been disconnected after hemispherectomy. Note the position of the functional right occipital lobe. **B** VEPs recorded from the left, middle and right occiput (occ). Pattern-reversal, pattern-onset and flash stimulation are shown. Note how symmetrical the pattern-reversal VEP responses are across the right and left occiput (blue arrows) despite the left occiput not being functional, this distribution was the same to a range of check sizes. Pattern-onset VEPs show the expected distribution being larger over the right occiput (green arrow) and smaller over the left occiput (red arrow).

Even in normal patients, the hemispheres are not symmetrical; there is a normal pattern of fronto-occipital asymmetry described with larger right than left frontal lobe and larger left than right occipital lobe [135]. This normal asymmetry in the occipital lobes has a consequence on the symmetry of the VEPs recorded at standard positions on the occipital scalp [131]. However, early-onset hemisphere pathology affects normal anatomical development of one hemisphere and its position in the skull. The affected hemisphere may be larger than the normal hemisphere (i.e., Hemimegalencephaly) and encroach on its space, or smaller than the normal hemisphere (i.e., atrophy after neonatal middle cerebral artery stroke) [136] leaving more space for the normal hemisphere to spread around posteriorly. These distortions in the anatomy will affect the orientation of the VEP generators, which in turn will affect the distribution of the trans-occipital asymmetries recorded over the scalp electrodes that we use in the diagnosis of uncrossed asymmetry/ a hemisphere defect and in turn suspected homonymous hemianopia (as seen in Fig. 12). The ISCEV standards [21] suggest the electrode channels O1, O2 and Oz for multichannel recordings. Though the standards and other studies show that additional lateral electrodes such as PO7 and PO8 or the Queens Square System can increase sensitivity [21, 88, 89, 116, 130, 134, 137]. More research is needed to investigate the applications of these larger electrode arrays in detecting hemisphere abnormalities in paediatric patients with distorted anatomy from early-onset hemisphere insults or congenital structural brain abnormalities.

More recently researchers have employed multifocal VEP methods to look for evidence of homonymous VF deficits in children. Multifocal VEP testing is challenging in children, due to the need to sustain central fixation throughout the test without breaks until the stimuli is over. However, some case reports and small case series have described its usefulness particularly in the investigation of VF defects in children being considered for epilepsy surgery [53, 138, 139].

OCT scans have been used in children to detect hemianopic visual-field deficits (both monocular and homonmous) using macular scans (as the loss resects the vertical midline) with reported sensitivity of 93% and specificity of 100% [7, 8] yet as previously discussed the time course to see these changes remains a limitation of this technique [11].

### Case 11: Homonymous hemianopia with typical VEP asymmetry

A 3 and a half-year-old girl was reviewed 2 months after surgical removal of myxoid astrocytoma of the suprasellar region with the right pterional approach. On her first post-operative MRI scan of the brain, damage of the right temporal lobe was described, which during subsequently at follow-up scans turned into stable hygroma. Her visual acuity was 0.15 (LogMAR) in each eye, confrontational VF was suspicious for left sided hemianopsia, she was turning the face to the left and both optic discs had sectors of pallor. She was not able to perform Goldmann perimetry and was referred for visual electrophysiology testing. Her findings are summarised in Fig. 11. Her flash ERG was normal, while her flash VEP showed abnormal trans-occipital amplitude distribution of the P2 and N2 waves. These were reduced over the right occiput and normal over the left occiput. The same lateralisation was observed for the C1 wave of the pattern-onset VEP, which was also larger over the left occiput. Pattern-reversal VEP also showed a significant trans-occipital asymmetry. However, due to the known paradoxical lateralisation, the P100 was largest on the right occipital electrode and significantly attenuated or reversed in polarity over the left occiput, indicating decrease of activity in the right occipital region. The electrophysiological findings of uncrossed asymmetry indicated left homonymous hemianopsia, so the girl was again referred to Goldman perimetry testing again, which showed suspicious left homonymous hemianopsia but was not reliable due to poor co-operation. Only at her visit, 2 and a half years later, Goldmann perimetry confirmed left homonymous hemianopsia.

### Case 12: Known homonymous hemianopia without typical VEP asymmetry

A 10-year-old girl presented with left hemimegalencephaly; a rare congenital disorder characterised by a maldevelopment and overgrowth of one hemisphere of the brain. The child had associated developmental delay and autistic spectrum disorder. The affected left hemisphere had been disconnected via hemispherectomy the previous year due to pharmaco-resistant life threatening seizures. As the left hemisphere had been disconnected, a right homonymous hemianopia was a certainty. Post-op VEPs were carried out as per department protocol. However, while pattern-onset VEP showed the expected lateralisation, this child’s pattern-reversal VEP was in fact symmetrically distributed (Fig. 12). This was reproducible to a range of tested check widths. The child’s co-operation was too poor to reliably perform half-field pattern VEP testing. The most likely explanation for this is due to the change in orientation of the calcarine fissure and as a result the pattern-reversal VEP generators. The pattern-onset VEPs demonstrated the expected pattern of trans-occipital asymmetry, being smaller over the affected left hemisphere. Even in normal patients the generators of pattern-reversal VEP are further within the calcarine sulcus than those of pattern-onset, which are more superficial [133]. Therefore, it is logical there might be a difference in lateralisation between the two types of pattern stimuli, as the anatomy of the calcarine sulcus is disturbed. Yet, the symmetrical pattern-reversal VEPs recorded from this patient with one functioning hemisphere would make it difficult to strongly suspect a homonymous hemianopia if we didn’t already know with certainty it was present. These marked distortions in the anatomy of the hemispheres are an example of why utilising clinical VEPs to look for evidence of suspected hemianopia in children with brain pathology can at times be inconclusive. In these cases sensitivity may well be improved when combined with MRI imaging studies.

## Conclusion

A range of visual-field defects may be found in paediatric patients, caused by a variety of retinal or post retinal pathology. Early diagnosis of visual impairment in childhood is important as it allows early intervention to maximise outcomes [140]. The assessment of the visual field in a child who is not able to undergo formal perimetry is a part of the paediatric ophthalmology clinic and often relies on a multi-disciplinary approach to build evidence. The role of electrophysiology testing in diagnosing and excluding a visual-field defects in pre-perimetric children is to differentiate between retinal disease, optic nerve, chiasm and hemisphere dysfunction, excluding non-organic vision loss. The test results can be used to evaluate the extent of the abnormality, monitor the progression and establish the prognosis of the disease. A detected electrophysiological abnormality can closely correlate or even precede the perimetric visual-field defect in some cases of peripheral VF loss [74, 85, 94, 95, 101]. However, VEPs might not be sensitive in all post-retinal cases of VF loss due to the dominance of the macular contribution to the pattern-reversal VEP, resulting in incomplete defects, macular sparing, or scotomas that do not affect the macular being undetected. Anatomical distortions of the visual cortex and the patient’s ability to fixate steadily on more complex stimuli (including nystagmus) can also reduce the sensitivity of electrodiagnostic tests in detecting VF defects caused by post-retinal pathology. Sensitivity to full-field stimuli decreases in post chiasmal pathology (Table 1). The use of multiple stimulation modalities, multichannel electrode arrays, as well as the employment of half- or quarter-field stimuli can improve the sensitivity (Table 1) [21, 88, 89, 94–98, 116–122, 127, 128, 130, 134, 137]. Therefore, careful protocol design is needed to maximise the sensitivity in these complex cases of post retinal pathology.

Much of the research in this area has been taken in adult subjects and so further work is needed in paediatric subjects who more reflect those we might see in clinical practice who cannot undertake perimetry. This work could explore the sensitivity and/or specificity of electrophysiological tests compared to other structural and functional techniques to investigate possible visual-field loss in these complex children. There is a potential role for deep learning to process large patient numbers from multiple centres. This would add to the evidence base that supports the decisions behind requesting different diagnostic tests in paediatric patients.

In children unable to complete formal perimetry testing visual electrophysiology is not a direct replacement, as it cannot provide the same level of detail of a visual-field defect such as depth, shape or position of scotoma. However, it remains a useful complementary technique to other testing (including OCT, fundus autofluorescence, fluorescein angiography, and MRI) to provide additional functional information increasing or decreasing the clinical suspicion of a visual-field defect in patients when formal perimetry is not possible.
